# Charge Redistribution and Spin Polarization Driven by Correlation Induced Electron Exchange in Chiral Molecules

**DOI:** 10.1021/acs.nanolett.1c00183

**Published:** 2021-03-24

**Authors:** Jonas Fransson

**Affiliations:** Department of Physics and Astronomy, Uppsala University, Box 516, 75121 Uppsala, Sweden

**Keywords:** chiral induced spin selectivity, electron−vibron coupling, exchange, spin-polarization

## Abstract

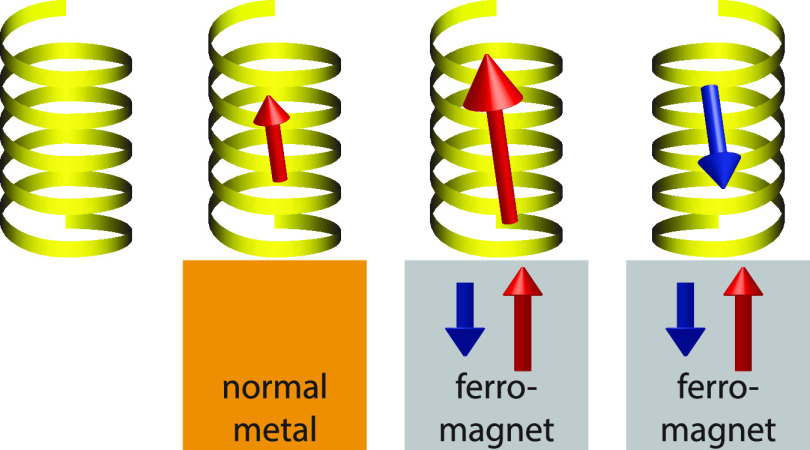

Chiral induced spin selectivity is a phenomenon that has been attributed to chirality, spin–orbit interactions, and nonequilibrium conditions, while the role of electron exchange and correlations have been investigated only marginally until very recently. However, as recent experiments show that chiral molecules acquire a finite spin-polarization merely by being in contact with a metallic surface, these results suggest that electron correlations play a more crucial role for the emergence of the phenomenon than previously thought. Here, it is demonstrated that molecular vibrations give rise to molecular charge redistribution and accompany spin-polarization when coupling a chiral molecule to a nonmagnetic metal. The presented theory opens up new routes to construct a comprehensive picture of enantiomer separation.

Since its discovery, chiral induced spin selectivity^1,2^ has been considered to emerge from the combination of structural chirality, spin–orbit interactions, and strongly nonequilibrium conditions. While chirality is a prerequisite, spin–orbit interactions are suggested to be one of the cornerstones in any theoretically comprehensible description.^3−28^ Nonequilibrium conditions, arising from the probing techniques used in the measurements, for instance, light exposure,^1,2,29−33^ local probing techniques,^34−37^ transport,^35,38,39^ and different types of Hall measurements,^30,31,40^ however, have typically not been regarded as part of the phenomenology. Particularly, in many theoretical considerations, nonequilibrium conditions have not been accounted for. Instead, the focus has lied on the transmission properties of chiral molecules embedded in a given environment.^3−13,15−18,20−22,24,25^ While the transmission pertains to the linear response regime, it is typically the result of a single particle description which, therefore, is not capable of resolving the chemistry or physics the molecule is subject to under nonequilibrium conditions.

In chemistry, it is well-known that addition or subtraction of one or several electrons can completely change the properties of the molecule. For instance, charge, as well as, spin polarization resulting from changing the number of electrons on the molecule may vary its intrinsic properties to a degree which can only be addressed in terms of sophisticated theoretical methods. Questions related to such structural changes were recently addressed,^23,41^ stressing the vital role of electronic Coulomb interactions in this context. It was shown that Coulomb interactions generate the exchange necessary for producing measurable effects regarding, for example, chiral induced spin selectivity and enantiomer separation. Other attempts along these lines, however, through electron-vibration^26,28^ and polarons,^27^ have shown the importance of expanding the theoretical concepts to more elaborate models.

In this context, it is also natural to question whether the chiral molecules maintain their intrinsically spin-degenerate properties when attached to metals. Indeed, recent experiments suggest that a strong spin-polarization can be associated with the interface between chiral molecules and metallic surfaces.^40,42−44^ In refs (40) and (42), chiral molecules were used to control the magnetism in a thin Co layer, resolved through the anomalous Hall effect. Enantiomer separation was concluded to be viable on nonmagnetic metals,^43^ whereas Yu–Shiba–Rusinov states^45−47^ were observed in the vicinity of chiral molecules on the surface of superconducting NbSe_2_.^44^ Since the observation of Yu–Shiba–Rusinov states is strongly related with the presence of localized magnetic moments, these results vividly suggest the emergence of finite spin moments when interfacing chiral molecules with metals. Related to these observations are also the results showing strongly enantiomer dependent binding energies on ferromagnetic metals.^48−51^ Enantiomer separation was addressed theoretically in ref (41). For molecules in contact with ferromagnetic metal, the electronic exchange plays a crucial role in the magnetic response. On the other hand, since these studies were made solely for molecules in an environment where the ferromagnet generates the symmetry breaking to which the electronic properties of the molecule respond, the question of whether chiral molecules themselves may generate a finite spin-polarization when in contact with a metal remains open.

A summary of the collected research, thus far, concerning the magnetic properties of chiral molecules is illustrated in Figure 1. In vacuum, the spin-degenerate charge is uniformly distributed in the molecule (Figure 1a). Upon coupling the molecule to a nonmagnetic metal, the charge is strongly redistributed resulting in a nonvanishing charge polarization (Figure 1b). By the chirality, the charge polarization is accompanied by a spin-polarization (Figure 1b). The intrinsic preference of the spin-polarization can be amplified or reduced with a ferromagnet (Figure 1c, d).

**Figure 1:**
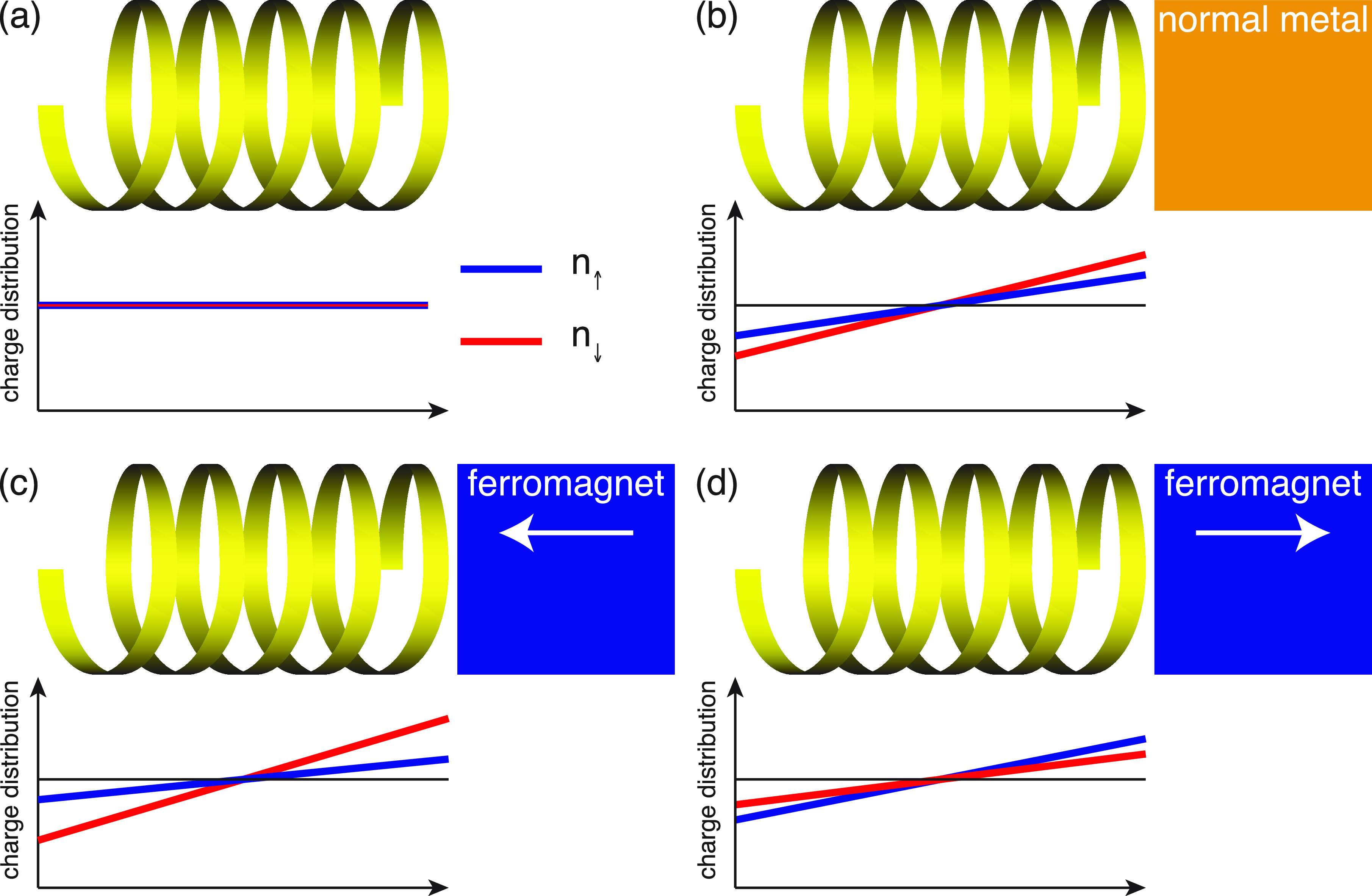
Chiral molecule in (a) vacuum, (b) in contact with nonmagnetic metal, (c, d) in contact with ferromagnetic metals with opposite magnetizations. The diagrams illustrate the spin resolved molecular charge distributions, *n*_*↑*_ (blue) and *n*_*↓*_ (red). The black lines indicate the charge distribution in a vacuum.

In this Letter, it is demonstrated that electron correlations originating in molecular vibrations is of crucial importance for the emergence of a finite spin-polarization in chiral molecules coupled to a metal. While the molecular structure is nonspin-polarized in vacuum, vibrationally assisted charge redistribution created in molecules attached to a metal generates a finite spin-polarization due to chiral induced charge–spin separation. The phenomenology is unequivocally shown to be associated with molecular vibrations combined with a strongly asymmetric charge polarization. Implementation of these results in a ferromagnetic environment corroborate, moreover, the importance of electron correlations as a source of exchange splitting between the spin channels.

The discussion presented here is based on simulations of idealized chiral models of realistic, e.g., α-helix, oligopeptides and polyalanines. Since the focus lies on the cooperation between chirality, spin–orbit interactions, and electron correlations, the mapping onto specific molecular compounds is less important as the details strongly vary between different structures. The model was proposed in ref (28). Using experimentally viable spin–orbit interaction parameters, it was shown, under *nonequilibrium* conditions, that the exchange splitting between the spin channels that was introduced by vibrationally supported correlations supports a chiral induced spin selectivity of tens of percents. The vibrationally supported correlation induced exchange splitting is, hence, a source for a substantial nonequivalence between the spin channels, a nonequivalence which is maintained under reversal of the magnetic environment.

A fundamental difference from essentially all previous theoretical studies is that, here the molecule is attached to a single metal and no external forces are applied. Hence, the molecule establishes a (quasi-)equilibrium state with the metal, in which no net charge current flows, which is the state considered here. While rapid transient evolution is fundamentally interesting in this context, it is beyond the scope of the present discussion. In this sense, the theory presented here is more directly appropriate for comprehending the results in, e.g., refs (40) and (42), while the connection is more loosely qualitative with the experiments reported in, e.g., refs (48−51).

The simulations are performed on a model of a chiral structure which constitutes a set of  ionic coordinates **r**_*m*_ = (*a* cos φ_*m*_, *a* sin φ_*m*_, *c*_*m*_), , and , where *a* and *c* define the radius and length, respectively, of the helical structure of *M* laps with *N* ions per lap. A Hamiltonian model can be written as
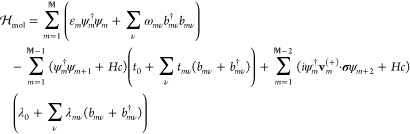
1Here, the molecule is described by a set of single-electron energy levels {ε_*m*_}, where ε_*m*_ denotes the energy level at the position **r**_*m*_, associated with the electron creation and annihilation spinors ψ_*m*_^†^ and ψ_*m*_, respectively. Nearest-neighboring sites interact, second line in eq 1, via direct hopping, rate *t*_0_, and electron–phonon assisted hopping, rate *t*_*mν*_. Similarly, the spin–orbit coupling is picked up between next-nearest neighbor sites, last line in eq 1, through processes of the type *iψ*_*m*_^†^**v**_*m*_^(*s*)^·**σ**ψ_*m*+2*s*_, *s* = ±1, where λ_0_ and λ_*mν*_ denote the direct and electron–phonon assisted spin–orbit interaction parameters, respectively, and where **σ** denotes the vector of Pauli matrices. The vector **v**_*m*_^(*s*)^ = **d̂**_*m*+*s*_ × **d̂**_*m*+2*s*_ defines the chirality of the helical molecule in terms of the unit vectors **d̂**_*m*+*s*_= (**r**_*m*_ – **r**_*m*+*s*_)/|**r**_*m*_ – **r**_*m*+*s*_|, and different enantiomers are, here, represented by different signs (±) of the chirality. The electrons are at each site coupled to the vibrational modes ω_*mν*_, which are represented by the phonon operators *b*_*mν*_ and *b*_*mν*_^†^, through the rates *t*_*mν*_ and λ_*mν*_. For simplicity and without loss of generality, each site is modeled to carry a single vibrational mode that couples to the electronic structure on-site only. By omitting intersite couplings, it is justified to assume that the ions vibrate with the same energy ω_0_ and that the on-site electron–phonon coupling parameters *t*_*mν*_ = *t*_1_ and λ_*mν*_ = λ_1_, for all *m* and ν. While the spin-independent coupling *t*_1_ is not strictly necessary to obtain results that are qualitatively similar to the one presented in the following discussion, it has been included since it is likely to be larger than the spin-dependent coupling λ_1_. Any Coulomb repulsion has been excluded since the vibrationally supported correlation induced exchange is expected to be dominating at room temperature, which is of main interest here.

The presence and properties of the metallic surface is captured by the parameter **Γ** = Γ_0_(σ^0^ + *pσ*^*z*^)/2, which represents the coupling between the ionic site *m* = 1 and the itinerant electrons in the surface. Here, Γ_0_ = 2*π∑*_**k**σ_|*v*_**k**σ_|^2^ρ_σ_(ε_**k**_) accounts for the spin-dependent hybridization *v*_**k**σ_ and spin-density of electron states ρ_σ_(ε_**k**_) in the metal, whereas |*p*| ≤ 1 denotes the effective spin-polarization of the coupling.

The properties of the electronic structure are related to the single electron Green function **G**_*mn*_(*z*) = ⟨⟨ψ_*m*_|ψ_*n*_^†^⟩⟩(*z*), letting **G**_*m*_ ≡ **G**_*mm*_, through, e.g., the density of electron states ρ_*m*_(ω) = *i*sp[**G**_*m*_^>^(ω) – **G**_*m*_^<^(ω)]/2π and spin resolved charges ⟨*n*_*mσ*_⟩ = (−*i*)sp(σ^0^ + σ_*σσ*_^*z*^σ^*z*^)∫**G**_*m*_^<^(ω)*dω*/4π, where **G**_*m*_^<(>)^ is proportional to the density of occupied (unoccupied) electron states. Here, sp denotes the trace over spin 1/2 space.

The equation of motion for the Green function **G**_*mn*_ = **G**_*mn*_(*z*) can be written on the form
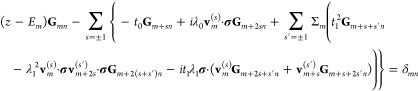
2Here, *E*_1_ = ε_1_ – *i***Γ**/2, which includes the level broadening due to the coupling **Γ** of this site to the metal, wheareas *E*_*m*_ = ε_*m*_, , and **G**_*mn*_ = 0 for . The self-energy Σ_*m*_ = Σ_*m*_(*z*) represents electron–phonon interaction loop described by
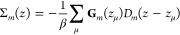
3where *z*_μ_ = *i*(2*n* + 1)π/β is the Fermionic Matsubara frequency, whereas β = 1/*k*_B_*T* is the inverse temperature *T* in terms of the Boltzmann constant *k*_B_. In terms of the simplest nontrivial electron–phonon interactions, the self-energy is given by
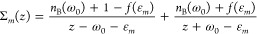
4where *n*_B_(ω) and *f*(ω) are the Bose–Einstein and Fermi–Dirac distribution functions, respectively. Insofar the exchange splitting generated by the molecular vibrations can be regarded as an intrinsic property of the structure, the employed approach is justified since it captures the main effect of the electron–phonon coupling. Hence, despite the charge redistribution, which may modify the exchange splitting, the gross effect of the electron–phonon interactions is captured by using the approximation of instantaneous thermalization of the vibrations.

The plots in Figure 2a show the charge distribution for the vibrating molecule mounted on the metallic surface. The charge distribution is at 300 K (i) strongly redistributed, with depleted charge in the interior of the molecule accumulating near the metal and (ii) accompanied by a nonvanishing spin-polarization; see Figure 2a, b. At low temperatures (20 mK), the charge is strongly confined to its bare electronic structure, since the vibrational excitations are thermally suppressed, which leads to a substantially weakened charge redistribution. Nevertheless, the small amount of charge reorganization that does occur is also accompanied by a nonvanishing spin-polarization, albeit much weaker than at elevated temperatures. The orientation of the emerging spin-polarization depends on the chirality of the molecules, Figure 2b, which is expected since only the chirality change upon shifting helicity from positive to negative. It should be noted that the absence of spin-polarization of the site adjacent the surface is an effect of the fixed boundary conditions. As reference these results are compared with the result of the static molecule, Figure 2c, and the vibrating molecule in vacuum, Figure 2d. In both configurations, the charge distribution is weakly nonuniform, and symmetric around the center of the molecule along its length direction, with vanishing spin-polarization, both at low and high temperatures. The charge variations in Figure 2c are due to the strongly delocalized nature of the electrons, such that the site index is not a good quantum number. Hence, the charge may accumulate or deplete nonuniformly throughout the structure while the total charge is conserved.

**Figure 2:**
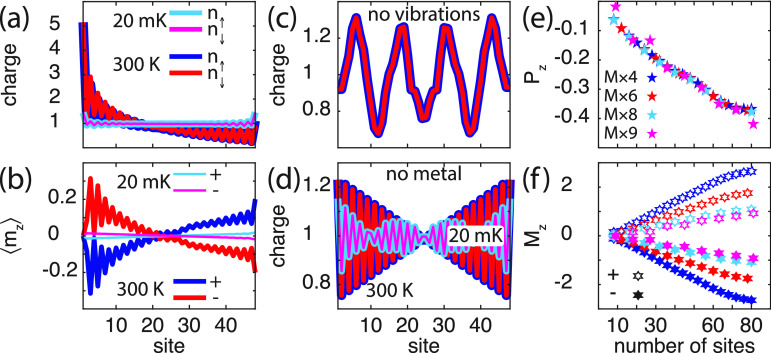
Chiral molecule (8 × 6) in contact with a metallic surface. (a, b) Spin-resolved charge distribution and corresponding spin-polarization (⟨*m*_*z*_⟩ = (*n*_*↑*_– *n*_*↓*_)/2) per site in the chiral molecule at the temperatures *T* = 20 mK and 300 K. In part a, the blue (cyan) and red (magenta) lines correspond to *n*_*↑*_ and *n*_*↓*_, respectively, at 300 K (20 mK). In part b, the blue (cyan) and red (magenta) lines represent positive (+) and negative (−) helicity, respectively, at 300 K (20 mK). (c) Spin-resolved charge distribution per site in the static chiral molecule at 20 mK (cyan, magenta) and 300 K (blue, red). (d) Spin-resolved charge distribution per site in the isolated vibrating chiral molecule at 20 mK (cyan, magenta) and 300 K (blue, red). (e, f) Charge and spin polarizations *P*_*z*_ and *M*_*z*_, respectively, as a function of the number of sites, at 300 K. (f) Positive (negative) helicity is denoted by open (filled) hexagrams. Here, parameters used in the simulations are, in units of *t*_0_ = 40 meV: ε_0_ – *E*_F_ = −2, Γ_0_ = 1/10, ω_0_ = 1/100, λ_0_ = 1/40, *t*_1_ = 1/10, and λ_1_ = 1/400, where *E*_F_ is the Fermi energy of the metal. An intrinsic broadening 1/τ_ph_ = *t*/4 was used in the vibration self-energy in order to smooth the electronic densities. In panel d, the long wavelength substructure is attributed to the numerical sensitivity to the integration mesh at low temperature.

The charge polarization, , , ⟨*n*_*m*_⟩ = *∑*_σ_⟨*n*_*mσ*_⟩, and , can be used for a normalized collective measure of the charge redistribution, such that |*P*_*z*_| ≤ 1. In this context, then, a negative (positive) charge polarization should be understood as a charge accumulation (depletion) in the end of the molecule adjacent to the metal, and charge depletion (accumulation) in the free end of the molecule (Figure 2a, c, d). In analogy to the charge polarization, a measure of the mean spin-polarization is given by *M*_*z*_ = 2*∑*_*m*_(*c*_*m*_ – ⟨*z*⟩)⟨*n*_*m↑*_– *n*_*m↓*_⟩/*L*. A negative (positive) value of this measure should, accordingly, be interpreted as an overweight of spin *↑* (*↓*) near the metal and/or an overweight of the opposite spin on the free end; see Figure 2b. Here, *P*_*z*_ and *M*_*z*_ associated with vibrating molecules at 300 K are plotted in Figure 2e, f, as a function of the molecule length. The plots demonstrate the growth of both charge and mean spin-polarization with molecular length. The charge polarization appears, in addition, to be a universal feature, as the values of the different types of molecules fall on essentially the same line. The deviations (*M* × 9) are an effect of the finite size which tend to vanish with increasing length. The absence of either vibrations or coupling to external environment leads to vanishing charge and mean spin-polarizations (Figure 2c, d). The results summarized in Figure 2 confirm that charge polarization and accompanied spin-polarization, in the composite system originates in electron correlations.

The above results demonstrate that a finite spin-polarization emerges in the chiral molecule when interfaced with a metal. Since the sign of the spin-polarization depends on the chirality, with an overweight of spin *↓* (*↑*) near the interface for positive (negative) helicity, this spin-polarization is expected to be diminished (enhanced) by a positive spin-polarization (*p* > 0) in the coupling parameter **Γ**. This expectation is corroborated in the spin-resolved charge distributions shown in Figure 3, for (a) positive and (b) negative helicity, and (c) corresponding spin-polarizations, in the setup with *p* = 0.1. Here, the spin-polarization of the molecule with positive helicity has a tendency to counteract the external spin-polarization, in a fashion which is not unlike a diamagnetic property. Opposite (negative) helicity tends, on the other hand, to magnetically act cooperatively with the external spin-polarization. The loss of mirror symmetry between the plots in Figure 3a and b is an expected outcome of the vibrationally supported correlation induced exchange. However, despite the molecular spin-polarization tends to be strongly modified by the external conditions, the intrinsic properties of the chiral molecules remain unchanged when comparing positive and negative helicity. This is shown by the difference ⟨*m*_*z*_⟩_+_ – ⟨*m*_*z*_⟩_–_ (helicity ±) in Figure 3d. The difference in the induced spin-polarizations is the same for any *p*, here shown for *p* = 0, 0.1, and 0.5, something which indicates an intrinsic anisotropy. Since a corresponding universal difference between the spin-polarizations does not exist in absence of the vibrationally generated correlations (not shown), this result shows a stability of the correlation induced spin-polarization. Moreover, while an external spin-polarization *p* tends to result in a slightly different charge polarization *P*_*z*_ for different helicity (Figure 3e), although minute, the mean spin-polarization *M*_*z*_ is strongly affected (Figure 3f), in accordance with the expected characteristics. The length dependence of the mean spin-polarization *M*_*z*_ is in good agreement with the results reported in, e.g., refs (2) and (48), especially for longer chains whereas the agreement is not as good for short chains.

**Figure 3:**
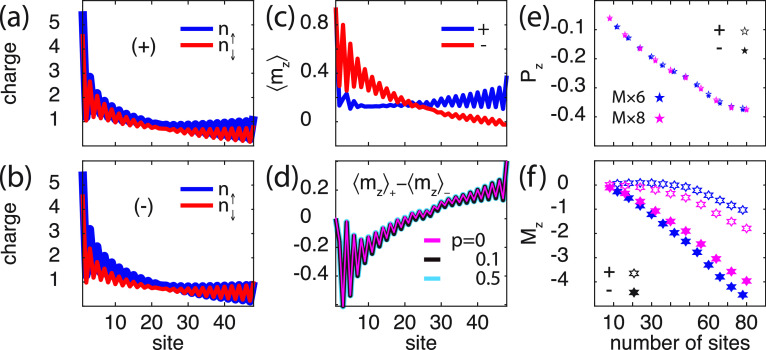
Chiral molecule (8 × 6) in contact with a ferromagnetic surface. (a, b) Spin-resolved charge distribution for a molecule with (a) positive and (b) negative helicity. (c) Corresponding spin-polarization per site in the chiral molecule, for (blue) positive (+) and (red) negative (−) helicity. (d) Difference ⟨*m*_*z*_⟩_+_ – ⟨*m*_*z*_⟩_–_ between the spin-polarizations for positive and negative helicity. (e, f) Charge and spin polarizations *P*_*z*_ and *M*_*z*_, respectively, as a function of the number of sites, for positive (open symbols) and negative (filled symbols) helicity. Here, *p* = 0.1 and *T* = 300 K, while other parameters are as shown in Figure 2.

The magnetic anisotropy generated by the chiral molecule can be shown to influence an external spin moment **S** which is coupled to the molecule via exchange *v*, modeled as *vψ*_1_^†^**σ**·**S**ψ_1_. Putting *p* = 0, such that the coupling **Γ** = Γ_0_σ^0^/2, ensures that the model describes a spin moment embedded in a nonmagnetic metal, corresponding to the setup in, e.g., ref (40). Self-consistent calculations with respect to the molecular charge distribution show that an initial spin moment , 0 ≤ φ < 2π, eventually reaches the final state ⟨**S**⟩ = ∓(0, 0, 1) for ± helicity (Figure 4a). This result demonstrates the existence of an intrinsically sustained anisotropy which can be coupled to and influence the magnetic properties of the environment. Here, the local moment is represented by a spin *S* = 1, for which the expectation value is provided by ⟨**S**⟩ = *∑*_α_⟨α|**S**|α⟩, with respect to the spin Hamiltonian  = *iv***S**·sp∫**σG**_1_^<^(ω)*dω*/4π.

**Figure 4:**
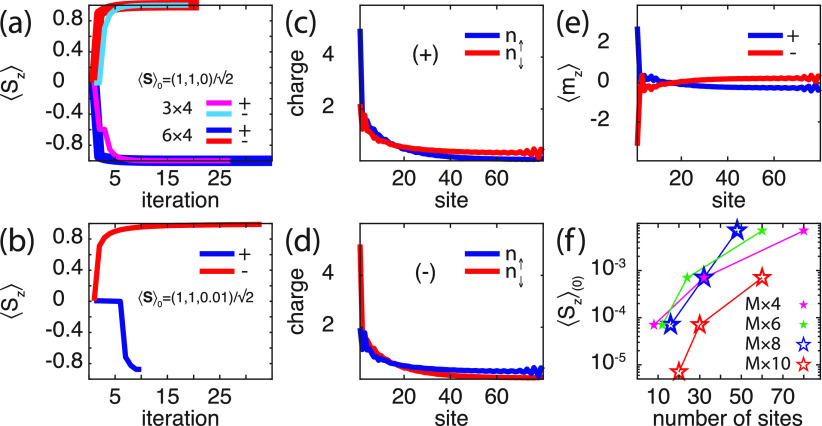
(a, b) Evolution of ⟨*S*_*z*_⟩ of the local spin moment **S** under the self-consistent simulations for (a) 3 × 4 and 6 × 4 molecules and (b) 20 × 4 molecule. (c, d) Spin-resolved molecular charge distributions in the self-consistent limit for (c) positive (+) and (d) negative (−) helicity. (e) Spin-polarizations ⟨*m*_*z*_⟩_±_ corresponding to the charge distributions in panels c and d. Maximum value of δ = ⟨*S*_*z*_⟩_0_ in the initial spin  that can be switched for different molecules characterized by *M* × *N*. Here, ε_0_ – *E*_F_ = −1/2, in panels c–f *t*_1_ = 1/20, λ_0_ = 1/10, λ_1_ = 1/20, *v* = 1 in units of *t*_0_, while other parameters and parameters in panel a are as shown in Figure 2.

By additionally exploring the intrinsic properties, adjusting the molecule parameters (see the caption of Figure 4 for details), it can, furthermore, be demonstrated that the intrinsic anisotropy is sufficiently strong to switch the spin moment from an initial ⟨*S*_*z*_⟩ = ±δ, 0 ≤ δ ≪ 1, to a final ⟨*S*_*z*_⟩ = ∓ 1 for ± helicity, which is shown in Figure 4b. The plots show the self-consistency evolution of the local moment  under the influence of a 20 × 4 molecule for positive (blue) and negative (red) helicity. The corresponding spin-resolved charge distributions for the molecules are shown in Figure 4c, d, and spin-polarization ⟨*m*_*z*_⟩ is shown in Figure 4e, demonstrating that the symmetry under the change of helicity is maintained also when the molecule is coupled to an external spin moment.

In the experimental observations of spin reversal using chiral molecules,^40,42^ the magnetization of a magnetized ferromagnetic layer, saturated in an out-of-plane configuration, was entirely switched. The simulations presented here show a weaker anisotropy associated with the magnetic properties of the composite system. The plots in Figure 4f display results of systematic simulations; all indicating an upper bound of δ, in the initial spin moment . Despite this limitation of the presented theory, it, nevertheless, shows that molecular vibrations act in the composite system as to generate strong magnetic anisotropies on externally located spin moments.

In summary, it has been shown that molecular vibrations in composite molecule–metal configurations are a mechanism that breaks the spin symmetry of the molecule, in accordance with experimental observations. While the vibrational source of exchange is particularly effective at high temperatures, it is non-negligible also at lower temperatures. It was, moreover, shown that this mechanism provides an origin for enantiomer separation using magnetic measurements. Temperature dependent anomalous Hall measurements may provide evidence for the vibrationally supported correlation induced exchange.
